# Akt/mTOR Role in Human Foetoplacental Vascular Insulin Resistance in Diseases of Pregnancy

**DOI:** 10.1155/2017/5947859

**Published:** 2017-09-14

**Authors:** Roberto Villalobos-Labra, Luis Silva, Mario Subiabre, Joaquín Araos, Rocío Salsoso, Bárbara Fuenzalida, Tamara Sáez, Fernando Toledo, Marcelo González, Claudia Quezada, Fabián Pardo, Delia I. Chiarello, Andrea Leiva, Luis Sobrevia

**Affiliations:** ^1^Cellular and Molecular Physiology Laboratory (CMPL), Division of Obstetrics and Gynaecology, School of Medicine, Faculty of Medicine, Pontificia Universidad Católica de Chile, 8330024 Santiago, Chile; ^2^Immunoendocrinology, Division of Medical Biology, Department of Pathology and Medical Biology, University of Groningen, University Medical Center Groningen (UMCG), 9700 RB Groningen, Netherlands; ^3^Department of Physiology, Faculty of Pharmacy, Universidad de Sevilla, 41012 Seville, Spain; ^4^Department of Basic Sciences, Faculty of Sciences, Universidad del Bío-Bío, 3780000 Chillán, Chile; ^5^Vascular Physiology Laboratory, Department of Physiology, Faculty of Biological Sciences, Universidad de Concepción, 4070386 Concepción, Chile; ^6^Institute of Biochemistry and Microbiology, Science Faculty, Universidad Austral de Chile, 5110566 Valdivia, Chile; ^7^Metabolic Diseases Research Laboratory, Center of Research, Development and Innovation in Health-Aconcagua Valley, School of Medicine, Faculty of Medicine, Universidad de Valparaíso, San Felipe Campus, 2172972 San Felipe, Chile; ^8^University of Queensland Centre for Clinical Research (UQCCR), Faculty of Medicine and Biomedical Sciences, University of Queensland, Herston, Brisbane, QLD 4029, Australia

## Abstract

Insulin resistance is characteristic of pregnancies where the mother shows metabolic alterations, such as preeclampsia (PE) and gestational diabetes mellitus (GDM), or abnormal maternal conditions such as pregestational maternal obesity (PGMO). Insulin signalling includes activation of insulin receptor substrates 1 and 2 (IRS1/2) as well as Src homology 2 domain-containing transforming protein 1, leading to activation of 44 and 42 kDa mitogen-activated protein kinases and protein kinase B/Akt (Akt) signalling cascades in the human foetoplacental vasculature. PE, GDM, and PGMO are abnormal conditions coursing with reduced insulin signalling, but the possibility of the involvement of similar cell signalling mechanisms is not addressed. This review aimed to determine whether reduced insulin signalling in PE, GDM, and PGMO shares a common mechanism in the human foetoplacental vasculature. Insulin resistance in these pathological conditions results from reduced Akt activation mainly due to inhibition of IRS1/2, likely due to the increased activity of the mammalian target of rapamycin (mTOR) resulting from lower activity of adenosine monophosphate kinase. Thus, a defective signalling via Akt/mTOR in response to insulin is a central and common mechanism of insulin resistance in these diseases of pregnancy. In this review, we summarise the cell signalling mechanisms behind the insulin resistance state in PE, GDM, and PGMO focused in the Akt/mTOR signalling pathway in the human foetoplacental endothelium.

## 1. Introduction

Insulin modulates D-glucose homeostasis, and a reduced response or a lack of response to this hormone (hereafter referred as “insulin resistance”) is characteristic in several pathologies, including diabetes mellitus and obesity [1, 2]. Insulin resistance tightly relates with abnormal responses of the vascular endothelium, that is, endothelial dysfunction, to vasoactive molecules including insulin and the endogenous nucleoside adenosine [3, 4]. Human pregnancy courses with physiological maternal and foetal insulin resistance as an adaptive response to the increasing nutrient requirement by the pregnant women and the growing foetuses [5].

Insulin signalling involves preferential activation of the protein kinase B (PKB)/Akt (Akt) and mitogen-activated protein kinase (MAPK) signalling pathways [4, 6]. Vascular actions of insulin in the human placenta and umbilical cord vessels (hereafter referred as “foetoplacental vasculature”) are of relevance since this vascular bed lacks innervation, and the control of the blood flux results from local release of vasoactive molecules [4, 7]. The mechanisms behind vascular insulin effects include the synthesis of nitric oxide (NO) by the endothelial NO synthase (eNOS) isoform, ATP release, and adenosine-mediated increase of L-arginine transport and NO synthesis [4, 8, 9]. Pathologies of pregnancy, such as preeclampsia (PE) [10] and gestational diabetes mellitus (GDM) [4, 11], and abnormal maternal conditions, such as pregestational maternal obesity (PGMO) and maternal obesity in pregnancy [12], show with reduced insulin signalling in the foetoplacental vasculature. In this review, we propose that common signalling mechanisms result in insulin resistance of the human foetoplacental vasculature in these diseases.

## 2. Insulin Signalling

**Figure 1:**
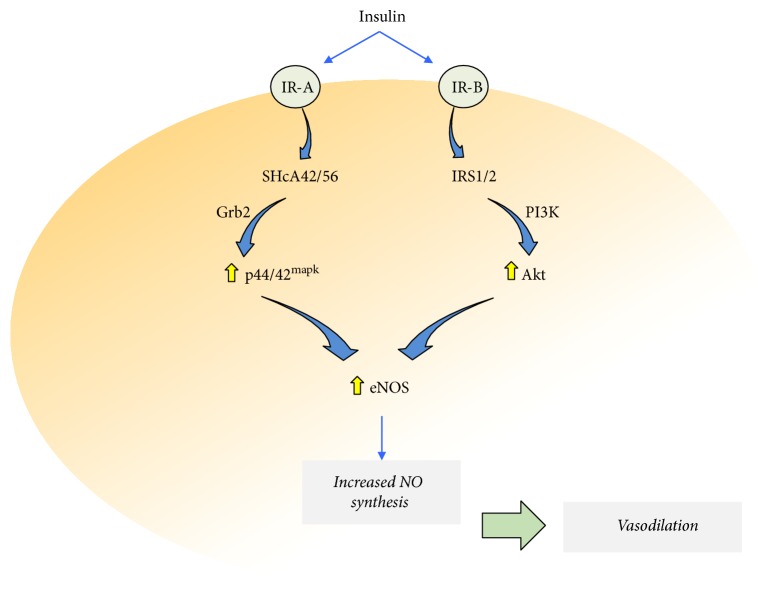
Insulin signalling in the human feotoplacental vasculature. Insulin activates insulin receptors A (IR-A) and B (IR-B) leading to recruitment and activation of insulin receptor substrates 1 and 2 (IRS1/2) and Src homology 2 domain-containing transforming protein 1 type A of 42 and 56 kDa (SHcA42/56). IR-A activation causes preferential activation of SHcA42/56, which triggers signalling through the growth factor receptor-bound protein 2 (Grb2) cascade ending in higher (⇧) activity of the 44 and 42 kDa mitogen-protein kinases (p44/42^mapk^). IR-B activation causes preferential activation of IRS1/2, which triggers signalling through the phosphatidylinositol 3 kinase (PI3K) cascade ending in higher protein kinase B/Akt (Akt) activity. IR-A signalling and IR-B signalling increase the endothelial nitric oxide (NO) synthase (eNOS) activity to generate nitric oxide (NO). An increase in the NO synthesis results in relaxation of the foetoplacental vascular beds (*vasodilation*).

Insulin activates the splice variants A (IR-A) and B (IR-B) of insulin receptors (IRs) in the human foetoplacental vasculature [13]. IR-A and IR-B are expressed in this vascular bed with IR-A showing higher affinity for insulin than that with IR-B [4, 13, 14]. IR activation by *β*-subunit autophosphorylation recruits and phosphorylates two protein families, that is, the insulin receptor substrates (IRSs) and the Src homology 2 domain-containing transforming protein 1 (SHc) [15] (Figure 1()). IRSs have at least six members (IRS-1 to IRS-6), where IRS-1 and IRS-2 are the most characterized [15]. SHc corresponds to at least three different proteins (SHcA, SHcB, and SHcC), with SHcA being expressed in mammals as the alternative splicing isoforms SHcA 46, SHcA 52, and SHcA 66 [16]. IRS-1 and IRS-2 are major activators of Akt via phosphatidylinositol 3 kinase (PI3K) compared with a minor effect on 44 and 42 kDa mitogen-activated protein kinases (p44/42^mapk^); instead, SHcA preferentially activates p44/42^mapk^ via the growth factor receptor-bound protein 2 (Grb2) [17]. However, whether stimulation of IR-A or IR-B results in differential SHc or IRS activation and signalling is unknown. The physiological response of most tissues in the human body, including the foetoplacental vasculature, is that activation of p42/44^mapk^ and Akt signalling pathways results in increased eNOS expression and activity leading to vasodilation [4, 18]. However, under pathological conditions, the equilibrium between signalling associated with IR-A and IR-B activation by insulin is lost and a preferential activation of p42/44^mapk^ or Akt is reported. Several studies describe a variety of cell signalling mechanisms potentially involved in these alterations of insulin response; however, upstream- and downstream-associated signalling pathways are not addressed.

## 3. Insulin Resistance

Insulin resistance is seen in subjects where the metabolic handling of D-glucose is deficient [2]. PE [19, 20], GDM [21, 22], and obesity in pregnancy [23] show with insulin resistance in the mother, foetus, and newborn. However, whether insulin resistance results from or is the cause of these pathological conditions is still unclear.

**Figure 2:**
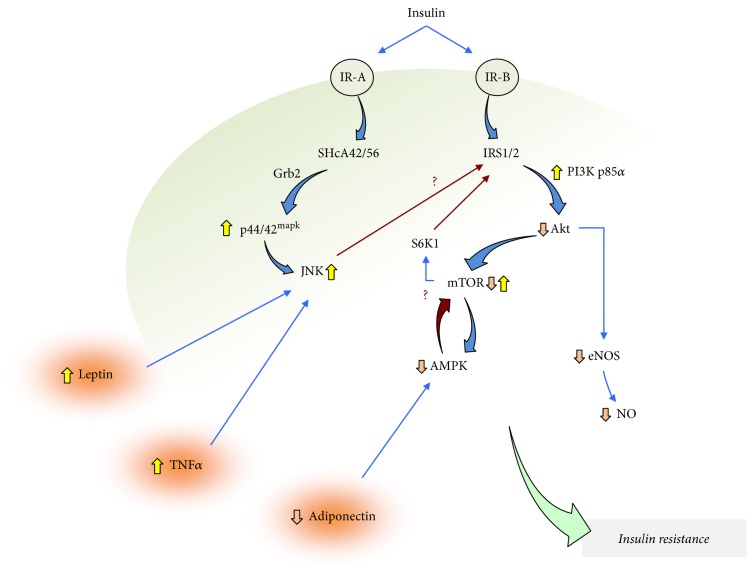
Cell signalling in insulin resistance in the human foetoplacental vasculature. Insulin activates insulin receptors A (IR-A) and B (IR-B) leading to recruitment and activation of insulin receptor substrates 1 and 2 (IRS1/2) and Src homology 2 domain-containing transforming protein 1 type A of 42 and 56 kDa (SHcA42/56). IR-A activation causes preferential activation of SHcA42/56, which triggers signalling through the growth factor receptor-bound protein 2 (Grb2) ending in increased (⇧) activity of the 44 and 42 kDa mitogen-protein kinases (p44/42^mapk^) and c-Jun N-terminal kinases (JNK). IR-B activation causes preferential activation of IRS^1/2^ triggering signalling by the p85*α* regulatory subunit of phosphatidylinositol 3 kinase (PI3K p85*α*). Activation of this subunit of PI3K decreases (⇩) the protein kinase B/Akt (Akt) activity ending in reduced endothelial nitric oxide (NO) synthase (eNOS) activity and NO generation. Reduced Akt activity also results in lower activity of the mammalian target of rapamycin (mTOR) activity, which turns into reduced activity of the adenosine monophosphate protein kinase (AMPK). Reduced AMPK activity is also caused by the reduced plasma level of adiponectin (an AMPK-activator) thus releasing AMPK-inhibition of mTOR facilitating activation of this molecule. This phenomenon potentially (?) increases mTOR-activated signalling through p70 S6 kinase 1 (S6 K1) thus reducing IRS1/2 signalling. The increased extracellular level of leptin and tumour necrosis factor *α* (TNF*α*) results in JNK activation. The possibility that JNK increases the inhibitor phosphorylation of IRS1/2 (Ser^312^) reducing insulin signalling (?) is likely. All in concert, these mechanisms lead to a state of lower response to insulin of the human foetoplacental vasculature (*insulin resistance*). Blue arrows denote activation. Red arrows denote inhibition.

Several studies show that IRS-1-mediated activation of PI3K leads to formation of phosphatidylinositol triphosphate, the substrate for the human 3-phosphoinositide-dependent protein kinase 1 (PDK1), which activates Akt [15] (Figure 2). However, in insulin resistance, IR-B preferential activation by insulin results in IRS1/2-mediated increase in the activity of the p85*α* regulatory subunit of PI3K (PI3K p85*α*), which inhibits Akt thus reducing NO generation. Other reports show that Akt activation mediates increased activity of the mammalian target of rapamycin (mTOR), a regulator of cell proliferation, adhesion, migration, invasion, metabolism, and survival [24]. Interestingly, mTOR signals through p70 S6 kinase 1 (S6K1) which reduces insulin signalling by inhibiting IRSs-activity-mediated activation of Akt [25, 26]. Thus, a modulatory loop to keep a physiological Akt activity and therefore insulin signalling to cause vasodilation involves mTOR activation/deactivation depending on the state of activation of Akt. When mTOR is upregulated, the physiological consequences are reduced Akt-mediated, NO-dependent vascular responses to insulin.

Other reports address that mTOR activity is inhibited by the adenosine monophosphate kinase (AMPK) [27], a molecule considered as general sensor of the cell energy state getting activated in response to a lower ATP/AMP ratio [28, 29]. AMPK activation results in increased eNOS activator phosphorylation at serine 1177 (Ser^1177^) and serine 615 (Ser^615^) in the vasculature [30]. Interestingly, AMPK activation increased the activity of PI3K/Akt/eNOS signalling cascade leading to higher NO generation and prevented the high D-glucose-impaired response to insulin in human umbilical vein endothelial cells (HUVECs) [31]. Thus, it is suggested that AMPK will increase insulin signalling due to its capacity to inhibit mTOR in the human foetoplacental vasculature.

Activation of p44/42^mapk^ triggers c-Jun N-terminal kinase (JNK) signalling in HUVECs, resulting in IRS inhibition [32, 33] (Figure 2). Since S6K1 activation by mTOR results in p44/42^mapk^- and Akt-reduced activity in HUVECs [34] and insulin-dependent activation of p44/42^mapk^ inhibits AMPK in the rat skeletal muscle cell line L6 [35], a functional dependency between p44/42^mapk^, AMPK, and mTOR activity may also be a phenomenon involved in impaired insulin sensitivity in the foetoplacental vasculature.

It is well described that proinflammatory cytokine tumour necrosis factor *α* (TNF*α*) [36] and the adipocytokine adiponectin [37] and leptin [38] play crucial roles in insulin resistance. TNF*α* activates the JNK signalling pathway in HUVECs [39] resulting in inhibition of IRS-1 and reduced Akt-mediated insulin signalling [40–42] (Figure 2()). Interestingly, higher plasma TNF*α* is found late in pregnancy (34–36 weeks of gestation) suggesting a likely reduced insulin biological action at this stage of pregnancy [43]. Adiponectin keeps insulin signalling (i.e., acts as insulin sensitizer) increasing the IRS-dependent signalling pathway by activating AMPK [37] and, subsequently, inhibiting mTOR [44]. Interestingly, a reduced plasma level of adiponectin is reported in pregnant women with diabetes mellitus [36]. Since the maternal plasma TNF*α* level is elevated in PE [45], GDM [46], or obese pregnant women [47], a potential TNF*α*-dependent inhibition of adiponectin release in insulin resistance in pregnant women, and perhaps the foetus, is likely. However, whether TNF*α* regulates adiponectin release in pregnancy is still unknown. Leptin is released in obesity in response to accumulating subcutaneous fat and increased fatty acid oxidation [38], a phenomenon regarded as a state of higher insulin resistance [38, 48]. Additionally, leptin activates JNK leading to inhibition of IRS1/2 and reduced insulin sensitivity [32, 33]. Since (i) leptin also increases the generation of reactive oxygen species (ROS) in HUVECs [49], (ii) superoxide anion (O_2_^−^), the most reactive ROS, scavenges NO [30], and (iii) ROS activates JNK in this cell type [49], a leptin/ROS (probably O_2_^−^)/JNK pathway is likely described as a mechanism leading to reduced insulin sensitivity in the human foetoplacental vasculature. Interestingly, increased leptin concentration in the maternal circulation is reported in GDM pregnancies [50, 51], a disease that also shows with increased ROS generation [9, 11]. Thus, this adipocytokine may also play a role in insulin resistance particularly in diseases of pregnancy where ROS generation is increased.

## 4. Insulin Resistance in Pregnancy Diseases

### 4.1. Preeclampsia

Preeclampsia (PE) is a heterogeneous pregnancy-specific multisystemic syndrome, defined by the occurrence of new onset hypertension (≥140/90 mmHg) and proteinuria (≥300 mg/24 hours) after 20 weeks of gestation [10, 52]. PE is of early onset (EOPE, <34 weeks of gestation) or late onset (LOPE, ≥34 weeks of gestation) [10, 53, 54]. EOPE and LOPE pregnancies associate with impaired insulin response of the maternal [55] and foetoplacental vasculature [20, 56]. However, not a clear mechanism explaining these alterations in EOPE and LOPE is yet available.

**Figure 3:**
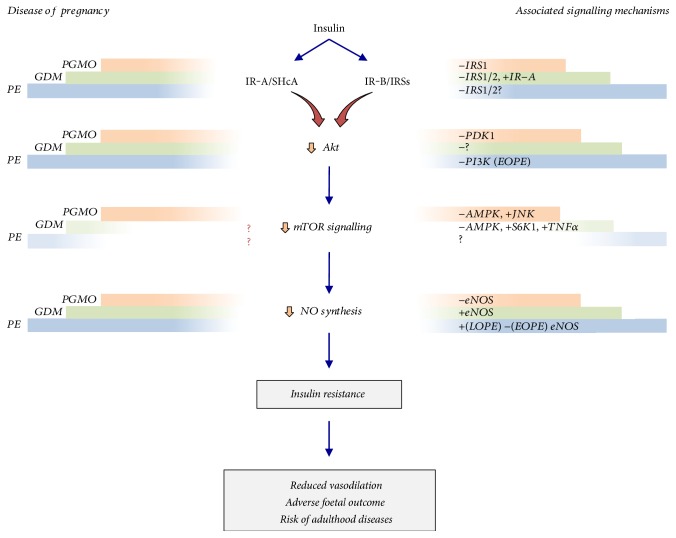
Potential involvement of Akt/mTOR in insulin resistance in the human foetoplacental unit from diseases of pregnancy. Pregestational maternal obesity (PGMO), gestational diabetes mellitus (GDM), and preeclampsia are diseases of pregnancy where the human foetoplacental endothelial function is reduced. The response of the placenta to insulin results from activation of insulin receptor A (IR-A) via preferential signalling through Src homology 2 domain-containing transforming protein 1 type A (IR-A/SHcA) and insulin receptor B (IR-B) via preferential signalling through insulin receptor substrates (IR-B/IRSs). The effect of PGMO (represented as orange bars), GDM (represented as green bars), and PE (represented as blue bars) in the cell signalling triggered by insulin causes an increase (+) or a decrease (−) in the expression and activity of the indicated associated signalling molecules for each pathology. The defective action of insulin is also documented for a reduced (⇩) activity of protein kinase B/Akt (Akt) due to signalling molecules that are reported for PGMO and early onset PE (*EOPE*), a phenomenon that is less clear (?) in GDM pregnancies. Reduced Akt activity results in reduced expression and activity of the mammalian target of rapamycin (mTOR) and its signalling in cells from PGMO, with a not clear mechanism (?) in GDM and PE. These changes result in reduced activation of the endothelial nitric oxide (NO) synthase (eNOS) activity leading to lower NO generation in PGMO and EOPE but increased eNOS activity in GDM and late onset PE (LOPE). These mechanisms lead to a reduced Akt/mTOR signalling cascade in response to insulin (*insulin resistance*) in the foetoplacental vasculature. This condition's outcome is a reduced vasodilation with several other adverse foetal outcomes and higher risk of developing adulthood diseases. PI3K: phosphatidylinositol 3 kinase; AMPK: adenosine monophosphate kinase; SK61: p70 S6 kinase 1; TNF*α*: tumour necrosis factor *α*; PDK1: human 3-phosphoinositide-dependent protein kinase 1; JNK: c-Jun N-terminal kinases. Specific signalling mechanisms for each molecule shown are described in the text. The magnitude of the bars represents the degree of involvement of the diseases of pregnancy at the corresponding mechanism.

Preferential activation of p42/44^mapk^ and Akt is described in the foetoplacental vasculature from PE. Preterm PE (<37 wg) with HELLP (Hemolysis, Elevated Liver enzymes, and Low Platelet count) courses with increased phosphorylated p42/44^mapk^ activation in villous trophoblast [57]. In addition, the maternal plasma level from women with EOPE shows a higher level of endothelin-1 (ET-1) [58], but reduced Akt activity in the placenta [59] (Figure 3). Thus, an ET-1-dependent inhibition of Akt reducing insulin signalling is likely in this disease. Furthermore, since Akt activity positively correlates with NO generation in human foetal endothelial cells [60], EOPE-associated foetoplacental vascular dysfunction due to reduced NOS activity may involve p44/42^mapk^/ET-1/Akt signalling. On the other hand, LOPE pregnancies show with unaltered p42/44^mapk^ [57] and unaltered [57] or decreased [61] Akt activity in the placenta. Intriguingly, eNOS protein abundance and activator phosphorylation (Ser^1177^) are higher in HUVECs from LOPE pregnancies [20], findings complemented by elevated nitrate/nitrite ratio in human umbilical vein serum [62, 63], but contrary to the reported lower nitrate/nitrite ratio [64] and NOS-generation of L-citrulline from L-arginine (index of NOS activity) [20] in this cell type. One plausible explanation for reduced NOS activity in HUVECs from LOPE pregnancies is a predominant functional effect of an increase of eNOS inhibitor (Thr^495^) compared with the effect of an activator (Ser^1177^) phosphorylation of this enzyme [20]. Earlier studies show increased IRS-1 (Ser^312^) and IRS-2 (Ser^731^) inhibitor phosphorylation in response to insulin in the placenta from LOPE pregnancies [65]. Since IRS1/2 are key activators of Akt, LOPE-reduced Akt and NOS activity could involve IRS1/2 inhibition. Thus, LOPE-associated impaired insulin response could result from reduced IRS1/2/Akt/eNOS signalling in the human foetoplacental vasculature. Since activation of mTOR results in reduced IRS1/2 activity, it is likely that this signalling molecule is involved in the effect of EOPE and LOPE on NOS activity. However, there is no information regarding the potential role of mTOR in the aetiology of EOPE or LOPE in this vascular bed.

Several reports support the involvement of circulating factors in the aetiology of PE including increased soluble Fms-like tyrosine kinase 1 (sFlt1), soluble endoglin (sEng), and reduced vascular endothelial growth factor (VEGF) plasma levels [66, 67]. The increased plasma levels of ET-1 and sEng result in a higher sFlt1 plasma level [68]. The latter reduces the availability of free VEGF-A to bind VEGF plasma membrane receptors and inhibition of PI3K/Akt signalling, including eNOS activity, in HUVECs [61, 69]. However, inhibition of the PI3K/Akt signalling does not alter sEng release from placenta explants or primary trophoblast in PE [59]; therefore, a differential response to PI3K/Akt-mediated insulin signalling in human foetoplacental endothelium versus trophoblast is likely. Interestingly, PI3K p85 phosphorylation at Tyr^688^ results in increased PI3K activity and Akt signalling in placental tissue from EOPE pregnancies [70]. The latter was proposed as a compensatory mechanism to the VEGF-reduced activation of PI3K/Akt signalling in this disease. However, PI3K p85 activator phosphorylation is unaltered in placentas from LOPE pregnancies [71], suggesting a different adaptive mechanism for insulin signalling in EOPE and LOPE pregnancies.

### 4.2. Gestational Diabetes Mellitus

GDM refers to any degree of glucose intolerance first recognized during pregnancy, diagnosed at 24–28 weeks of gestation [2]. GDM associates with maternal obesity [72] and high risk of the mother to develop T2DM [73]. GDM presents with clinical manifestations in the mother [74], foetus [75, 76], and newborn [75, 77], including hyperglycaemia and hyperinsulinemia (see also [78, 79]). It is reported that IR-A expression and insulin receptor *β*-subunit (*β*-IR) activity are increased in HUVECs from GDM [80] (Figure 3). Interestingly, the ratio for p44/42^mapk^/Akt is >1 due to increased p44/44^mapk^, but unaltered Akt activity, suggesting preferential activation of IR-A in this cell type. However, reduced IR-A, but increased IR-B expression, with a p44/42^mapk^/Akt ratio < 1 was reported in human placental microvascular endothelium. Insulin restored IR-A and IR-B expression and p44/42^mapk^/Akt ratio suggesting differential activation of insulin signalling cascades due to differential activation of IR subtypes in the macrovascular and microvascular foetoplacental endothelium from GDM pregnancies.

GDM associates with reduced uptake of the endogenous nucleoside adenosine, a potent vasodilator in most tissues, including the foetoplacental vasculature [4]. This phenomenon results in elevated extracellular concentration of adenosine enough to activate adenosine receptors [81], preferentially A_2A_ adenosine receptors (A_2A_AR), in the foetoplacental endothelium from GDM pregnancies [4, 11]. Interestingly, GDM also increases hCAT-1-mediated L-arginine transport in HUVECs [82], which seems to link with an increased eNOS activity and NO synthesis in this cell type. The latter study also shows that insulin reversed the GDM-increased L-arginine transport requiring A_1_AR activation. Thus, different adenosine receptors are involved in the modulation of L-arginine transport in HUVECs from normal compared with GDM pregnancies.

AMPK activation is lower in the placenta from women with GDM [83, 84]. This finding is complemented by high levels of TNF-*α* and activation of NF-*κ*B, conditions leading to increased synthesis of mediators of inflammation and impaired insulin action [85, 86]. Thus, reduced AMPK expression could associate with a proinflammatory state and insulin resistance in GDM pregnancies. Since AMPK inhibits mTOR activity [27, 44], a reduced AMPK activation could result in increased mTOR activity in GDM. GDM also courses with hyperleptinemia in the placenta [87, 88] and reduced adiponectin level [89] in umbilical vein plasma. However, precise mechanisms at insulin signalling in this disease are unclear.

Insulin treatment of women with GDM (i.e., patients under insulin therapy) reverses the GDM-associated maternal and foetal hyperglycaemia and the increase in IRS-1 and PI3K p85*α* activity caused by this disease to values in normal pregnancies [90]. However, the elevated level of leptin in the foetal plasma and TNF-*α* and IL-1*β* levels in the placenta from GDM pregnancies were unaltered by insulin therapy. Thus, insulin therapy results in normalization of foetal and maternal glycaemia but does not restore the impaired insulin signalling in foetoplacental endothelium in this disease. Indeed, we recently reported that insulin therapy in women with GDM did not restore the increased L-arginine uptake and NO synthesis seen in HUVECs from women with GDM under a controlled diet [91]. It is worrying that a higher chance to be born large for gestational age is reported as an outcome for insulin therapy [92] or in pregnant women treated with insulin and metformin [93] and in a larger number (~25%) of infants showing one or more episodes with neonatal morbidity where neonatal asymptomatic hypoglycaemia was the most frequent [94]. We emphasize our call regarding the still unclear effect of maternal insulin therapy on foetus development, the newborn, and postnatal life [2, 4, 9, 91, 95].

### 4.3. Pregestational Maternal Obesity

The World Health Organization defines obesity as individuals with a body mass index (BMI) > 30 kg/m^2^, a disease that has reached epidemic characteristics worldwide [1]. One of the main risks of an abnormal nutritional state is its association with metabolic syndrome, a condition with high multiple risk factors for chronic diseases, including diabetes mellitus, cardiovascular diseases, stroke, hypertension, and cancer [96].

Few studies address cell signalling in PGMO. Epidemiological evidence shows that children born to PGMO pregnancies show hyperinsulinemia and elevated insulin resistance [97, 98]. Additionally, infants and adolescents from PGMO pregnancies exhibit high risk of developing obesity [99, 100] and associate with higher cardiovascular risk in adulthood [100]. Interestingly, umbilical cords from PGMO pregnancies show a gene profile related with reduced insulin sensitivity [101], including downregulation of *PDPK1* (coding for PDK1) involved in D-glucose uptake and storage [101]. However, direct functional evidence for insulin effect on foetoplacental endothelium in PGMO is limited (Table 1).

PGMO pregnancies associate with reduced activity of AMPK [102] but increased activity of mTOR [103] in the placenta. These findings correlate with reduced maternal plasma adiponectin levels [104]. Since JNK activation is also increased in human placentas from PGMO pregnancies [105], a potential insulin resistance condition resulting from IRS inhibition may involve adiponectin-reduced level-dependent AMPK inactivation, increased mTOR activity, and reduced Akt signalling, in this abnormal condition of pregnancy (Figure 3).

## 5. Concluding Comments

Insulin regulates canonical signal transduction pathways initiated by activation of IR-A/p44/42^mapk^ and IR-B/Akt in human foetoplacental vasculature in healthy pregnancies (Figure 3). IRS-1 and IRS-2 are upstream activators of the PI3K/Akt signalling pathway leading to activation of mTOR. SHcA 42 and SHcA 56 activate p44/42^mapk^ leading to increased release of vasoconstrictors, such as ET-1. Insulin resistance associated with PGMO, PE, and GDM results in foetoplacental vascular dysfunction and altered vascular reactivity to insulin. A likely potential common point in insulin resistance in these diseases is a reduced Akt signalling resulting in lower activation of mTOR and eNOS. A role for AMPK in this phenomenon is not clear, but the involvement of this molecule is likely since its activation positively correlates with mTOR activity. A role of NO in the response to insulin in the foetoplacental endothelium in diseases of pregnancy is well described [4, 10, 12]. Thus, modulation of NO generation could be a final target of an abnormal IR-A/SHcA/p44/42^mapk^- and IR-B/IRSs/Akt-mediated signalling via Akt/mTOR in insulin resistance at the human foetoplacental vasculature. A therapy targeting these signalling molecules could be beneficial to improve insulin response in these diseases. PGMO is a risk factor for developing PE [106, 107] and GDM [107]. Thus, characterizing potential common signalling mechanisms for PGMO, PE, and GDM will facilitate the design of an approach to prevent insulin resistance in the co-occurrence of these or other disorders in pregnancy, thus reducing or abolishing their deleterious consequences for the mother, the foetus, and the newborn.

## Figures and Tables

**Table 1:** Effect of pathologies of pregnancy on insulin signalling in the human foetoplacental vasculature.

Cell or tissue	Molecule or activity	Effect of the pathology	Effect of insulin	References
*Preeclampsia*				
Placenta (EOPE)	p44/42^mapk^	Increase	*na*	[57]
Placenta (EOPE)	ET-1, ET_A_, and ET_B_ (mRNA)	Increase	*na*	[108]
Placenta	Akt-Ser^473^	Decrease	*na*	[61]
Placenta	eNOS	Increase	*na*	[109]
Placenta (LOPE)	*β*-IR, IRS-1-Tyr^465^, IRS-1-Ser^312^, and IRS-2-Ser^731^	No effect	Increase	[65]
Placenta (LOPE)	Akt-Ser^473^	No effect	Increase	[110]
HUVECs (LOPE)	eNOS-Thr^495^, eNOS-Ser^1177^	Increase	Restored	[20]
HUVECs (LOPE)	eNOS-Ser^1177^	Increase	*na*	[111]
HUVECs (EOPE)	eNOS	Decrease	*na*	[111]
HUVECs	eNOS	Decrease	*na*	[112]
HUVECs (LOPE)	L-Arginine transport	Increase	Restored	[20]
HUVECs (LOPE)^∗^	hCAT-1	Increase	Increase	[20]
*Gestational diabetes mellitus*				
Placenta	IRs	Increase	*na*	[113]
Placenta (insulin therapy)^∗∗^	*β*-IR	Increase	Restored	[90]
Placenta	IRS-1	Increase	*na*	[113]
Placenta (insulin therapy)	IRS-1	Increase	Restored	[90]
Placenta (insulin therapy)	IRS-2	Increase	Increase	[90]
Placenta	PI3K p85*α*	Increase	Restored	[90]
Placenta	PI3K p85*α*	Increase	*na*	[113]
Placenta (insulin therapy)	PI3K p110	Increase	No effect	[90]
Placenta^∗∗∗^	mTOR-Ser^2448^, S6K1-Thr^421^/Ser^424^	Increase	*na*	[83]
Placenta^∗∗∗∗^	S6 K1-Thr^389^, 4EBP1-Thr^37/46^	Increase	*na*	[114]
Placenta^∗∗∗^	4EBP1-Thr^37/46^	Increase	*na*	[83]
Placenta	AMPK (mRNA)	Decrease	*na*	[88]
Placenta	Adiponectin	Decrease	*na*	[115]
Placenta	TNF-*α*	Increase	*na*	[85, 116]
Placenta (insulin therapy)	TNF-*α*	Unaltered	*na*	[86]
Placenta	IL-1*β*	Increase	*na*	[116]
Placenta	Leptin receptor	Increase	*na*	[88]
Trophoblast	Leptin receptor	Increase	*na*	[87]
HUVECs	IR-A (mRNA)	Increase	Restored	[21]
HUVECs	Akt-Ser^473^	No effect	Increase	[80]
HUVECs	eNOS, eNOS-Ser^1177^	Increase	Restored	[80]
HUVECs	p44/42^mapk^-Thr^202/204^	Increase	Restored	[80]
HUVECs (insulin therapy)	eNOS, eNOS-Ser^1177^	Increase	Restored	[117]
HUVECs	hENT1, adenosine transport	Decrease	Increase	[21, 80]
HUVECs	L-Arginine transport	Increase	Restored	[82]
HUVECs (insulin therapy)	L-Arginine transport	Increase	Restored	[117]
hPMECs	p44/42^mapk^-Thr^202/204^, Akt-Ser^473^	Decrease	Restored	[118]
hPMECs	IR-A (mRNA)	Decrease	Restored	[118]
hPMECs	IR-B (mRNA)	Increase	Restored	[118]
hPMECs	hENT1	Decrease	No effect	[118]
hPMECs	hENT2	Decrease	Restored	[118]
hPMECs	hENT1 transport activity	Decrease	No effect	[118]
hPMECs	hENT2 transport activity	Decrease	Restored	[118]
Umbilical cord plasma	Leptin	Increase	*na*	[88]
Umbilical cord plasma	Adiponectin	Decrease	*na*	[89]
*Pregestational maternal obesity*				
Placenta	AMPK-Thr^172^	Decrease	*na*	[102, 103]
Placenta	AMPK	Decrease	*na*	[103]
Placenta	S6 K1-Thr^389^	Increase	*na*	[88, 119]
Placenta	JNK-Thr^183^/Tyr^185^	Increase	*na*	[119]
Placenta	mTOR (mRNA)	Decrease	*na*	[102, 103]
Placenta	IRS-1 (mRNA)	Decrease	*na*	[103]

AMPK: adenosine monophosphate protein kinase; AMPK-Thr^172^: AMPK phosphorylated at threonine 172; S6K1: S6 kinase 1; S6K1-Thr^421^/Ser^424^: S6K1 phosphorylated at threonine 421 and serine 424; S6K1-Thr^389^: S6K1 phosphorylated at threonine 389; JNK: c-Jun N-terminal kinases; JNK-Thr^183^/Tyr^185^: JNK phosphorylated at threonine 183 and tyrosine 185; mTOR: mammalian target of rapamycin; IRS-1: insulin receptor substrate 1; IRS-1-Tyr^465^: IRS-1 phosphorylated at tyrosine 465; IRS-1-Ser^312^: IRS-1 phosphorylated at serine 312; IRS-2: insulin receptor substrate 2; IRS-2-Ser^731^: IRS-2 phosphorylated at serine 731; EOPE: early-onset preeclampsia; LOPE: late-onset preeclampsia; p44/42^mapk^: 44 and 42 kDa mitogen-activated protein kinases; p44/42^mapk^-Thr^202/204^: p44^mapk^ phosphorylated at threonine 202 and p42^mapk^ phosphorylated at threonine 204; Akt: protein kinase B/Akt; Akt-Ser^473^: Akt phosphorylated at serine 473; eNOS: endothelial nitric oxide synthase; eNOS-Thr^495^: eNOS phosphorylated at threonine 495; eNOS-Ser^1177^: eNOS phosphorylated at serine 1177; IRs: insulin receptors; IR-A: insulin receptor A; IR-B: insulin receptor B; *β*-IR: insulin receptor *β*-subunit; PI3K: phosphatidylinositol 3 kinase; PI3K p85*α*: p85*α* regulatory subunit of PI3K; PI3K p110: p110 catalytic subunit of PI3K; EGFR: epidermal growth factor receptor; mTOR-Ser^2448^: mTOR phosphorylated at serine 2448; S6K1-Tyr^389^: S6K1 phosphorylated at threonine 389; 4EBP1: eukaryotic translation initiation factor 4E binding protein 1; 4EBP1-Thr^37/46^: 4EBP1 phosphorylated at threonine 37 and 46; TNF-*α*: tumour necrosis factor *α*; AP1: activator protein 1; NF-*κ*B: nuclear factor-kappa B; ET-1: endothelin 1; ET_A_: endothelin receptor type A; ET_B_: endothelin receptor type B; IL-1*β*: interleukin 1*β*; hCAT-1: human cationic amino acid transporter 1; hENT1: human equilibrative nucleoside transporters 1; hENT2: human equilibrative nucleoside transporters 2; HUVECs: human umbilical vein endothelial cells; hPMECs: human placental microvascular endothelial cells. ^∗^Cells incubated with insulin in the presence of ZM-241385 (A_2A_AR antagonist). ^∗∗^GDM mothers were obese. ^∗∗∗^Results include GDM mother under diet and insulin therapy. ^∗∗∗∗^GDM mother on oral insulin-sensitizing antidiabetic undefined medication. *na*: not assayed.
